# Timing of extreme heat events matters: exposure during parasitism disrupts top-down control

**DOI:** 10.1007/s00442-025-05781-6

**Published:** 2025-08-12

**Authors:** Nicholas A. Pardikes, Tomas A. Revilla, Gregoire Proudhom, Melanie Thierry, Chia-Hua Lue, Jan Hrcek

**Affiliations:** 1 Biology Centre of the Czech Academy of Sciences, Institute of Entomology, Ceske Budejovice, Czech Republic; 2 Department of Biology, Utah State University, Logan, UT USA; 3 Faculty of Science, University of South Bohemia, Ceske Budejovice, Czech Republic; 4 Centre de Recherche Sur La Biodiversité Et L’Environnement (CRBE), UMR 5300 CNRS-IRD-TINP-UT3, Université Toulouse III-Paul Sabatier, Toulouse, France; 5 Department of Biology, Hood College, Frederick, MD USA

**Keywords:** Climate change, Host–parasitoid, Communities, Ontogeny, Tropical ecology

## Abstract

Due to ongoing climate change, extreme climatic events are expected to increase in magnitude and frequency. While individual species’ responses to thermal extremes are widely studied, the impact of extreme heat events on species interactions and the key functions they provide in communities is understudied. As outcomes of species interactions depend on coordinated physiology and development, the consequences of heat exposure are likely impacted by its timing relative to the organisms’ life history traits, but to what extent is unclear. In this study, we evaluate how the timing of heat exposure affects interactions among nine tropical *Drosophila*–parasitoid species combinations using laboratory microcosm experiments. Interactions were most affected when heat exposure coincided with parasitism, leading to decreased parasitism rates. Parasitism rates also dropped when extreme heat occurred after parasitism in one instance. Experiencing heat exposure before parasitism had little effect. Using a simulation model, we determined that the combined effects of parasitism and heat exposure are generally additive, with no evidence of delayed consequences of heat exposure early in development on parasitism outcomes. Furthermore, we found adult host flies and parasitoids more resistant to heat exposure than their larval stages. Thus, whether more frequent extreme heat events disrupt species interactions globally will depend on their exact timing relative to ontogenetic stages and interactions. Heat exposure impacts the two trophic levels differently. Thus, when heat exposure coincides with parasitism, it may diminish the ability of parasitoids to control their hosts, affecting both natural ecosystems and agricultural environments.

**Supplementary Information:**

The online version contains supplementary material available at 10.1007/s00442-025-05781-6.

## Introduction

Climate change has continued to intensify over the past two decades (IPCC 2023; Harvey et al. 2023). In addition to increasing mean temperatures, the frequency and severity of extreme events such as heatwaves, droughts, and heavy precipitation have risen, transforming ecosystems and species interactions globally (Pachauri et al. 2015). Ectotherms, which comprise a significant portion of Earth’s biodiversity, are particularly vulnerable to extreme temperatures, because they rely on external temperatures to regulate physiological processes (Huey et al. 1999; Kingsolver et al. 2011; Harvey et al. 2020; Ma et al. 2021). In addition, a non-linear relationship between performance and temperature in ectothermic organisms means that even slight increases in temperature at high temperatures can have significant adverse physiological effects (Dillon et al. 2010; Paaijmans et al. 2013; Sinclair et al. 2016). Thus, extreme heat is predicted to impact ectotherm survival, growth, reproduction rates, population dynamics, and interactions with other organisms (Kingsolver and Huey 2008; Boukal et al. 2019; González‐Tokman et al. 2020). At the community level, species with different thermal sensitivities may respond differently to heat exposure, leading to trophic mismatches and altered interaction strengths (Kaspari et al. 2015; Buckley and Huey 2016). Thus, as temperatures continue to increase and become more variable, most organisms are likely to experience extreme heat at some point in their lifetime, even those with short lifespans. While the immediate physiological responses of individual organisms to heat exposure have been documented, it is unclear how brief but intense heat affects species interactions and the functions they provide in communities and ecosystems.

The effect of heat exposure on species interactions is difficult to predict due to differences in thermal sensitivity among species (Kaspari et al. 2015; Buckley and Huey 2016), but data for interacting species in communities and food webs are rare (but see Moore et al. 2021). In addition, species interactions are typically coordinated among the developmental stages of interacting species, and thermal sensitivity often changes as individuals grow and mature (Bowler and Terblanche 2008; Kingsolver et al. 2011; Zhang et al. 2015a; Zhao et al. 2017; Liu et al. 2024). As a result, the ecological effects of heat exposure likely vary depending on ontogenetic stage (Zhang et al. 2015b; Cinto Mejía and Wetzel 2023; Cope et al. 2023). While previous studies have highlighted the impacts of different heat exposure timings on individual species interactions (Agosta et al. 2018; Chen et al. 2019b, a; Valls et al. 2020; Moore et al. 2021; Costaz et al. 2023; Malinski et al. 2023; Parker and Kingsolver 2024), examining multiple interactions within a community is rare.

Species interactions between hosts and parasitoids offer an excellent opportunity to examine the impact of the timing of extreme heat on ecological communities, as their interactions are ubiquitous in nature, and their outcomes are easily quantifiable (Malinski et al. 2024). In addition, parasitoids play an important role in regulating the dynamics of host populations globally, and studies focusing on increased average temperatures rather than extreme temperatures have demonstrated that temperature can modify the outcome of host–parasitoid interactions (Hance et al. 2007; Meisner et al. 2014; Furlong and Zalucki 2017). Parasitoids, usually wasps or flies, lay their eggs inside or on an insect host, leading to the host’s death, and they are crucial for top-down regulation of lower trophic levels (Godfray 1994). Parasitoid performance is closely linked to temperature at each life stage, affecting adult activity, oviposition, and larval development. Short-term temperature extremes may change host resistance and parasite virulence, influencing the interaction’s outcome and limiting the ability to regulate abundances of lower trophic levels. In general, parasitoids have short adult lifespans and high fecundity (Godfray 1994). These traits may make them particularly susceptible to extreme temperatures, as adult females must rapidly locate and parasitize hosts during a narrow window of viability, with limited capacity for delayed reproduction or recovery from exposure (Zhang et al. 2019). Understanding how the timing of extreme heat events intersects with such tightly constrained reproductive strategies is essential for predicting the persistence of parasitoid populations under future climates.

Besides the thermal stress caused by extreme heat, parasitism events are inherently stressful for hosts, as they must expend energy to fight against parasitoid infection (Godfray 1994). Thus, the combined effects of heat exposure and parasitism on host mortality may act independently (i.e., additively) or with higher order effects (i.e., synergistically or antagonistically) (Todgham and Stillman 2013). If additive, mortality due to heat exposure and parasitism would act independently, and the combined impact on host abundance would be the sum of the effects of the two stressors in isolation. If higher order effects are acting, heat exposure may alter the ability of hosts to resist infection, resulting in either higher or lower mortality rates than expected based on additive effects. Given the short duration of extreme heat events, insect hosts may have time to recover from potential heat damage during later stages of development (Zhang et al. 2015a). However, the extent to which this varies across different heat exposure timings relative to host–parasitoid interaction and host and parasitoid species remains unclear.

Here, we experimentally test the impact of the timing of heat exposure relative to parasitism events in a tropical host–parasitoid community using laboratory microcosms. Using nine *Drosophila*–parasitoid species combinations, we assess whether the timing of heat exposure alters host mortality and parasitism outcomes. We also explore whether the combined stress of heat exposure and parasitism impacts host mortality through additive or higher order effects, using a single-generation, parameterized mathematical model based on experimental results. In a second experiment, we measure fly and parasitoid adult survival when exposed to the same brief but extreme heat to compare vulnerability across life stages.

## Materials and methods

### Study system

We focused on a host–parasitoid community of *Drosophila* and their parasitoid natural enemies from mid-elevation tropical forests in North Queensland, Australia (Jeffs et al. 2021; Pardikes et al. 2022). North Queensland’s tropical forests have been experiencing extreme heat events, but few studies have investigated how these systems respond to such extreme events despite predictions that tropical species may be more sensitive to extreme temperatures than temperate systems (Deutsch et al. 2008; Huey et al. 2012; Nairn and Fawcett 2017; Trancoso et al. 2020). We used three coexisting *Drosophila* species, *D. birchii, D. bipectinata, and D. simulans*, and three undescribed parasitoid wasp species, *Asobara* sp. (Braconidae: Alysiinae), *Ganaspis* sp. (Figitidae: Eucoilinae), and *Leptopilina* sp. (Figitidae: Eucoilinae) from the same community (Lue et al. 2021). All three parasitoid species are undescribed, and their life histories are still being determined. The three parasitoid species are solitary koinobiont parasitoids, where the host continues to develop and feed, while the parasitoid larvae are developing within and only one parasitoid wasp emerges from a single host infection. Based on other species from the same genera, we assume that *Leptopilina sp.* and *Ganaspis sp.* are pro-ovigenic, meaning that most of their eggs are fully developed when they first emerge as adult parasitoids. In contrast, *Asobara sp.* is likely synovigenic, as its eggs continue to mature during the parasitoid’s lifetime as an adult (Jervis et al. 2001). These parasitoids can live as adults up to 1 month at 24* °C* in the lab, but their lifespan in nature is unknown.

All fly and wasp lines used in this experiment were collected and established from the tropical forest of Paluma (*S19°00.386′ E146°12.732′*) Mountain Range in North Queensland, Australia in 2017 and 2018. They were then maintained in the Hrcek lab at the Czech Academy of Sciences (Ceske Budejovice, Czech Republic) in a 12L:12D photoperiod at a constant 24 *°*C. The parasitoids used in this experiment were maintained on *D. melanogaster* to avoid establishing any a priori preference for the three *Drosophila* species used. The experiment was performed in the summer of 2019. Before this experiment, eight isofemale lines from each fly species were combined to reintroduce genetic diversity and create a mass-bred line for each species. These mass-bred lines were then transferred into plastic population boxes (47 cm-length x 30 cm-width) × 27.5 cm-height) with mesh-covered openings for ventilation. One hundred milliliter of fresh fly medium was added to each box (2 boxes/fly species) three times a week to increase their abundance for the experiment. One month prior to the experiment, all *Drosophila* and parasitoid species involved in this study were moved from a constant 24 °C chamber to one with a 12L:12D photoperiod, 80–90% humidity, and a temperature regime that mirrored natural ambient conditions to acclimatize them to variable temperatures (Online Resource 1: Figure S1). All parasitoids received honey water before being placed in each experimental vial and during the 24-h foraging period.

Developmental rates vary among the three *Drosophila* species and are strongly influenced by temperature, with egg-to-adult development taking 8–16 days at a constant temperature of 24 °C. All three parasitoid species attack the larval stages of the three *Drosophila* species. However, based on previous observations, not all hosts or larval instars are of equal quality. For instance, *D. bipectinata* is a low-quality host for all three parasitoid species, but it remains susceptible to infection from each, which decreases its survival rates (Thierry et al. 2021). Importantly, host larvae are not viable hosts throughout their entire larval stage, as there is an infection window during which parasitism events can lead to the complete development and emergence of adult wasps from the infected host (Pardikes et al. 2022). All three larval parasitoids prefer late 1st and 2nd instar *Drosophila* larvae, which corresponds to 2–5-day-old larvae, depending on the species of *Drosophila*.

### Experiment 1: effects of timing of heat exposure on host–parasitoid interactions

Using a fully factorial design, we investigated the immediate and extended effects of the timing of extreme heat exposure on the outcome of host–parasitoid interactions in a laboratory experiment. The factors were host species (three levels: *D. birchii, D. bipectinata, *and* D. simulans*), parasitoids (four levels: no parasitoid, *Asobara* sp.*, Ganaspsis* sp., *Leptopilina* sp.), and extreme temperature timing (four levels: no heat, heat-before, heat-during, heat-after). All factors were fully crossed, resulting in 48 unique treatment combinations. Each combination consisted of six replicates initiated over 3 days, represented as blocks in our statistical analyses.

We first determined the average host development times (i.e., egg-to-adult) and host survival in the absence of heat exposure and parasitoids among the three *Drosophila* species. While still excluding parasitoids, we exposed *Drosophila* larvae to a simulated short-term extreme high temperature event for 24 h at three developmental periods relative to a host–parasitoid interaction (i.e., before, during, and after). The *heat-before* treatment started on the same day the fly eggs were introduced into the vials, subjecting the eggs and very early instar larvae to intense heat. The *heat-during* treatment exposed 3-day-old *Drosophila* larvae to 24 h of extreme high temperatures, while 7-day-old *Drosophila* larvae experienced a 24-h exposure to extreme heat in the *heat-after* treatment. Thus, any difference in *Drosophila* growth rates and survival was due solely to the timing of heat exposure.

Next, we investigated treatments in which parasitoids infected *Drosophila* larvae at ambient temperature regime among the nine different host–parasitoid pairs. Three mated female parasitoids were introduced to vials containing 3-day-old larvae and were allowed to forage for 24 h, after which all parasitoid wasps were removed. If any female parasitoids died during this time, we recorded the number of surviving adult females still alive. This allowed us to assess how parasitoids affect host survival without extreme heat.

Finally, we examined the nine host–parasitoid species combinations subjected to the three simulated 24-h extreme heat event timings. The *heat-before* treatment exposed the fly eggs and very early instar larvae to intense heat-before parasitism occurred. The *heat-during* treatment exposed 3-day-old *Drosophila* larvae to extremely high temperatures simultaneously with the 24-h foraging period of parasitoids. In the *heat-after* treatment, 7-day-old *Drosophila* larvae, previously exposed to parasitoids, experienced 24 h of heat exposure. Exposing 7-day-old larvae after parasitism helped us assess whether extreme heat alters the interaction’s result by either weakening the host’s immune defenses, thereby boosting parasitoid success, or damaging the recently injected parasitoid larvae or eggs, resulting in reduced success. This design also enabled us to test whether parasitism affects the host’s ability to cope with heat stress, leading to higher host mortality. This fully factorial design allowed us to separate the effects of heat exposure timing from the impact of parasitism on host survival. We were also able to examine how brief but intense temperature spikes affect *Drosophila* and parasitoid species at various developmental stages, recognizing that variation in the timing of extreme heat will impact different ontogenetic phases. All experiments were conducted using glass vials with a diameter of 2.8 cm and a height of 9 cm, and each vial contained 10 ml of fly medium. Fifty *Drosophila* eggs for a given host species were added to each vial (Nouhaud et al. 2018). The number of emerging flies and parasitoids was recorded daily. Collection stopped after four consecutive days of no emerging adult flies or parasitoids. We understand that subjecting *Drosophila* and parasitoids to extreme heat in glass vials limits their chances of moving to more favorable microclimates, which are typically found in natural situations. Thus, our results may be more extreme than those that occur in nature due to a lack of ability to avoid the extreme temperatures via microclimates.

Ambient temperature treatments had an average temperature of 20 °C with a low of 16 °C and a high of 24 °C, while the heat exposure treatments had an average of 28 °C with a low of 20 °C and a high of 34 °C. Both temperature regimes included two 4-h ramping periods (Online Resource 1: Figure S1). The flies and wasps experienced high daily temperatures for 4 h during the 24-h period and the low temperature for 12 h. This cycle attempted to mimic representative temperature patterns within a day. Based on temperature loggers placed in the field in Australia, the ambient temperature regime corresponds to the annual average daily temperature at mid-elevation study sites. The heat exposure regime was selected based on the long-term climate data from weather stations near each collection site and matched extreme temperatures recorded under rainforest canopies. For example, at the Paluma mid-elevation location, 34 °C was only measured once over 2 years of data collection. An important aspect of extreme heat events typically not included in studies is the associated increase in nighttime temperatures. Therefore, we decided to increase the maximum daily and nighttime temperatures to better represent natural conditions. We thus prioritized realism over a more theoretical alternative of varying just amplitude and keeping the mean daily temperature constant. The experiment occurred in a 12-h light/12-h dark photoperiod that matched the ramping periods (06:00–18:00 and 18:00–06:00). We used four growth chambers, two for each regime, to avoid pseudoreplication (Hurlbert 1984).

### Using simulations to identify combined effects of heat exposure timing and parasitism

We ran simulations to investigate if the interaction between parasitoid attacks and timing of extreme heat exposure leads to higher order effects on host survival. Under the null hypothesis that combined effects of parasitism and heat exposure do not include higher order effects (synergistic or antagonistic), the expected total number of flies F Eq. (1) and wasps W Eq. (2) emerging at the end of day T = 8 of the experiment and after are, respectively:

1 $${\text{F}} = {\text{H}}_{0} \,\exp ( - {\text{m}}_{0} {\text{T}} - {\text{m}}_{\tau } )\exp ( - {\text{a}}_{0} {\text{P}})$$

2 $${\text{W}} = \varepsilon \,{\text{H}}_{0} \exp ( - {\text{m}}_{0} {\text{T}} - {\text{m}}_{\tau } )[1 - \exp ( - {\text{a}}_{0} {\text{P}})]$$

where “exp” denote exponential functions. The equations are a variant of the Nicholson–Bailey host–parasitoid model (Bailey 1931), modified for single-generation experiments started with H_0_ = 50 eggs. A fraction “exp(-m_0_T -m_τ_)” of H_0_ develops during 8 days with baseline mortality (m_0_) plus differential mortality (mτ) on heat exposure day τ. Hosts avoid infection by P = 3 female parasitoids on day 3 with probability “exp(-a_0_P)” (a_0_ is attack rate per wasp) and become flies. The infected fraction “1-exp(-a_0_P)” begets wasps with probability ε per host. The product of exponentials in Eq. 1 leads to additive mortalities “m_0_T + m_τ_ + a_0_P” (baseline + heat exposure + parasitism), and the three are assumed independent, i.e., no higher order effects.

We used fly emergence data from controls to estimate baseline mortalities (m_0_) and mortality differentials (m_τ_) caused by heat exposure on day τ = 1, 3, or 7 (before, during, or after attack). Wasp emergence at control temperature was used to fit attack rates (a_0_) and wasps per infected host (ε). These parameters were used to project fly and wasp numbers emerging at the end of day 8, with a stochastic implementation of Eqs. 1 and 2. If the distribution of simulated and experimental data overlaps, the null hypothesis of additive effects of heat exposure and parasitism is not rejected. If they do not overlap, higher order effects between parasitism and timing of heat exposure are acknowledged. Model derivation, parameter fitting, and simulations are detailed in Online Resource 2. Calculations were done with MATLAB (The MathWorks Inc. 2022) and see Online Resource 3 for more details.

### Experiment 2: heat exposure tolerance of adult flies and parasitoid wasps

We quantified adult heat exposure tolerance of hosts and parasitoids in a second experiment. They were host species (three levels: *D. birchii, D. bipectinata, D. simulans*), parasitoid species (three levels: *Asobara* sp.*, Ganaspis* sp., *Leptopilina* sp.), and heat exposure (two levels: no heat, heat exposure). We randomly selected 20 male and 20 female adult flies or wasps 2 days after emergence for the heat exposure and ambient temperature treatments. Male and female adults were placed into separate vials with agar and assigned to their respective treatments. Adults remained for 24 h in each treatment. We counted the number of flies or wasps living after the treatment and quantified the proportion of adults that survived the 24-h heat exposure. All treatments were performed in the same thermal chamber and time block.

### Response variables

We analyzed the development times of *Drosophila* hosts across different heat exposure treatments using data from vials with no parasitoids. Development time refers to the duration from when eggs were placed into the vial until they emerged as adults. We also recorded the total count of adult *Drosophila* (di) and adult parasitoids (pi) that emerged from each vial. Host survival (HS) proportion was calculated using HS = di/T, where T was the average number of adult flies reared for a given *Drosophila* species in a corresponding temperature treatment without parasitoids following Pardikes et al. 2022 (Online Resource 1: Table S1). Thus, survival rates below 100% indicate that fewer adult flies survived the heat exposure treatment with wasps compared to those in the same treatment without wasps. This is a standard analysis of host–parasitoid interactions, so the decline in host survival is directly linked to parasitoids (Carton and Kitano 1981; Boulétreau and Wajnberg 1986; Stacconi et al. 2015). In cases where HS > 1 (more flies emerged in the presence of parasitoids compared to control treatments without parasitoids), we set HS = 1. Similarly, the parasitism rate (PR) was calculated using PR = pi/T. T represents the previously described value and indicates the maximum number of flies that can serve as a host for the parasitoid for a specific host species and heat exposure timing treatment. In cases where PR > 1 (more parasitoids emerged than flies in control treatment), we set PR = 1. Thus, differences in HS or PR across heat exposure timings were due to the impact of heat exposure on parasitoids or host defenses, not host availability, which varied by heat exposure timing and species and was analyzed separately.

### Statistical analyses

All statistical analyses were performed using R statistical software R version 4.2.1 (R Core Team 2022). We included all three factors (host species, parasitoid treatment, and heat exposure timing treatment) as fixed factors in all our models. From a total of 14,400 initial larvae, 5,537 flies and 2,870 parasitoids successfully emerged (Online Resource 1: Tables S2 and S3). Our statistical analyses aimed to quantify the impact of heat exposure on host–parasitoid interactions from both the host and parasitoid perspectives. We used a linear regression model to estimate development times across heat exposure treatments, host species, and potential interactions between the two factors as fixed effects. Log-transforming development time data improved model fit and helped meet assumptions of normality. We found no evidence that temperature treatments varied with the chamber’s location (Online Resource 1: Figure S2).

We used a binomial Generalized Linear Mixed Model (GLMM) with a logit link function to determine the proportion of host survival (HS) and parasitism rates (PR). All models included the fixed effects of heat exposure timing treatment, host species, parasitoid treatment, chamber location, and block as predictors. We removed *D. bipectinata* from all models for our PR model, because we reared only eight adult parasitoids, all from the *Ganaspis* sp. parasitoid (Online Resource 1: Tables S2 and S3). We included the *D. bipectinata* findings in the HS models, because parasitoids were clearly infecting *D. bipectinata* larvae, which lowered fly survival rates. However, only a limited number of these infections led to the emergence of adult wasps. We used a mixed model approach with an observation-level random effect (OLRE) to account for overdispersion and heteroscedasticity in our binomial data. All models included a three-way interaction between heat exposure timing, host species, and parasitoid treatments. Including the number of surviving wasps (out of three) after 24 h access to *Drosophila* larvae as a covariable did not improve models and was removed from all subsequent analyses.

To analyze adult fly survival, we used a binomial Generalized Linear Model (GLM) with a logit link function to determine the proportion of adult flies that survived. We used a GLMM for adult parasitoid survival with an OLRE to meet the assumption of overdispersion. To determine whether the effects of heat exposure on survival varied with species, our model included treatment and its interaction with species. In addition, we included sex as a factor to account for any differences in heat exposure tolerance between males and females, but found no evidence that extreme heat tolerance varied between sexes. The interaction between species and heat exposure treatment was not significant in both models, and the additive model was used to estimate the effects of heat exposure on adult survival rates.

The *car* package was used to identify significant predictors through Type III analysis of Deviance (Fox et al. 2015). To ensure that assumptions of normality, non-constant error variance, and overdispersion were not violated, we used the *DHARMa* package (Hartig 2019). We averaged over block and heat exposure chamber locations to calculate the estimated marginal means. We performed post-hoc multiple comparisons using Wald chi-squared tests in the *emmeans* package and adjusted *p* values using the Sidak method (Lenth et al. 2025). All figures were generated using the ggplot2 package, and coefficient tables were generated using the *sjPlot* package (Wickham 2011; Lüdecke et al. 2024).

## Results

### Impacts of heat exposure timing on Drosophila hosts

We examined development times and survival rates without parasitoids to determine which *Drosophila* species were thermally stressed by heat exposure. We expected that development would slow down if temperatures exceeded thermal optima, and survival rates would decrease. Based on survival and development times, 24 h of high temperature exposure were stressful for *D. birchii* but not for *D. bipectinata* or *D. simulans* (Online Resource 1: Figures S3 and S4, Tables S4 and S5). Compared to larvae in ambient temperatures, 7-day-old *D. birchii* larvae exposed to high temperatures had 7.7 times lower odds of surviving to adulthood (OR = 0.13, SE = 0.033, *P* < 0.0001). The greatest differences in developmental rates were observed when 7-day-old *D. birchii* larvae experienced high temperatures (Online Resource 1: Figure S3). *D. birchii* took 4% longer to develop. In comparison, *D. bipectinata* and *D. simulans* developed 7% and 6% faster, respectively.

### Effects of heat exposure timing on host–parasitoid interactions

**Fig. 1:**
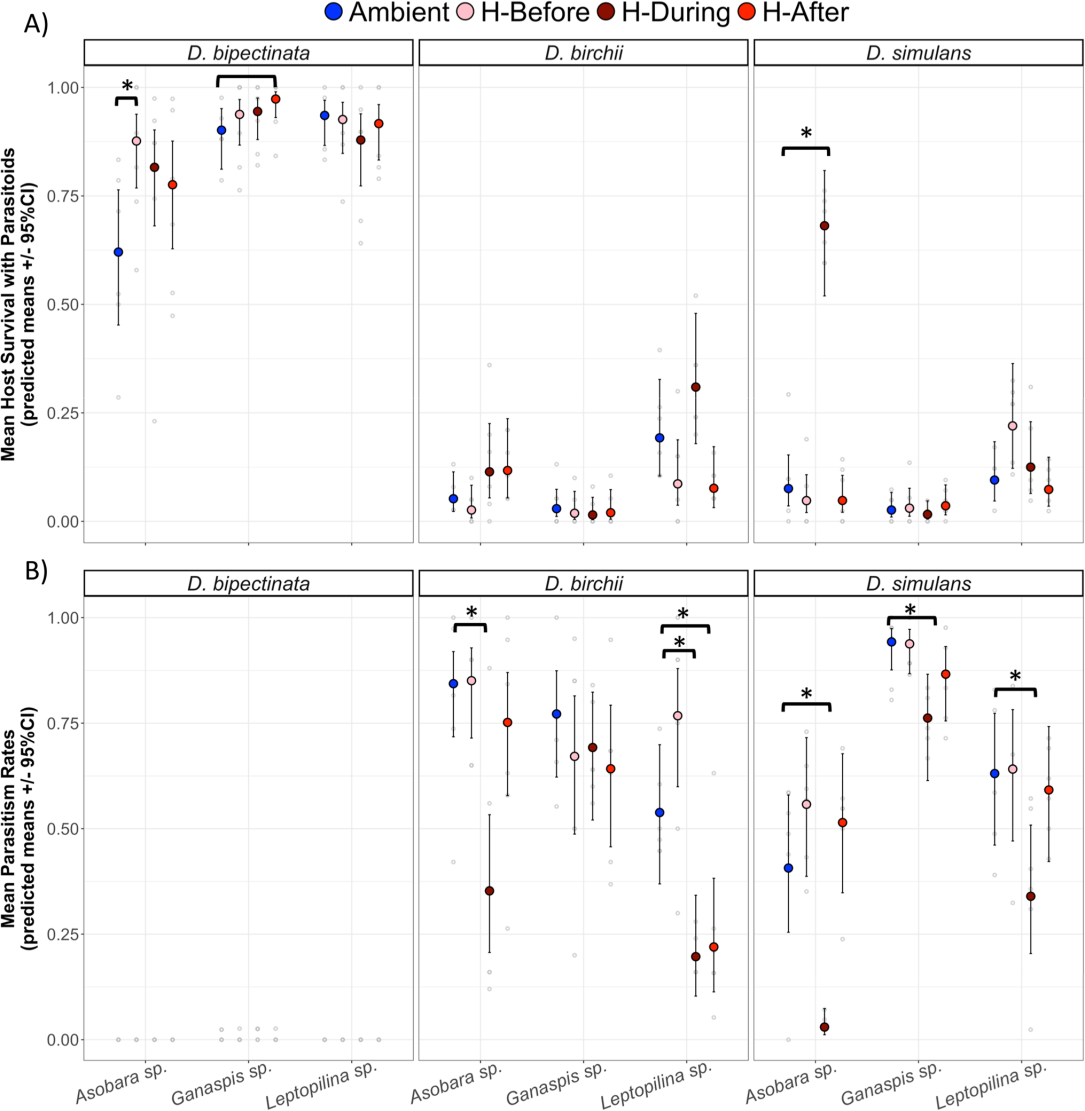
**A** Host survival when exposed to parasitoids and **B** parasitism rates at different heat exposure timings. Colored points represent (**A**) the estimated number of flies that emerged relative to the number of emerged flies not exposed to parasitoids and (**B**) the estimated number of parasitoids that emerged relative to the number of flies that emerged in control treatments without parasitoids. Both are averaged across chamber locations and blocks. The host *D. bipectinata* was dropped from the (**B**) parasitism rate analysis due to few emerging parasitoids. Stars indicate significant comparisons (*p* < 0.05), and the plain black bar represents a marginally significant comparison (*p* < 0.1) when compared to the ambient temperature treatment tested on the log odds ratio scale. Grey points are the observed (**A**) proportion of host survival and (**B**) parasitism rate for each replicate

The effect of heat exposure on host–parasitoid interactions can be comprehensively evaluated by considering both the host and parasitoid perspectives. We first report the host perspective (host survival), followed by the parasitoid perspective (parasitism rate). The impact of heat exposure timing on host survival rates varied significantly among host–parasitoid species combinations (Fig. 1A; Online Resource 1: Table S6). Only two of the nine unique combinations showed significant differences among the three heat exposure timing treatments and the control, and both involved the parasitoid species *Asobara* sp. Heat exposure during *Asobara* sp. parasitism increased the odds of *D. simulans* survival by 26 times compared to control temperatures (OR = 26.15 ± 13.9 SE, *p* < 0.0001). *D. bipectinata* larvae were 4.3 times (OR = 4.3 ± 2.25, *p* = 0.014) more likely to survive to the adult stage in the presence of *Asobara* sp. if the heat exposure occurred before the parasitism event. When averaged across all host and parasitoid species, heat exposure during parasitism events increased the odds of host survival by 1.7 times (OR = 1.7 ± 0.35 SE, *p* = 0.03) compared to ambient temperatures.

The effect of heat exposure timing on parasitism rates varied significantly across host and parasitoid species (Fig. 1B; Online Resource 1: Table S7). Five of the six analyzed host–parasitoid species combinations showed a significant decrease in parasitism rates when heat exposure occurred during the parasitism event. For example, the odds of successful parasitism by *Asobara* sp. were 10 and 20 times lower than those never exposed to high temperatures when attacking *D. birchii* (OR = 0.10, SE = 0.05, *p* = 0.001) and *D. simulans* (OR = 0.05, SE = 0.03, *p* < 0.0001), respectively (Fig. 1B). In addition, in a single case, the odds of parasitism declined by 4.2 times when the heat exposure followed parasitism (OR = 0.24, SE = 0.13, *p* = 0.02, *Leptopilina* sp. on *D. birchii*). On average, if heat exposure occurred during or after parasitism, the odds of successful parasitism were reduced by 5.5 (OR = 0.18, SE = 0.04, *p* < 0.001) and 1.7 times (OR = 0.58, SE = 0.13, *p* = 0.03), compared to ambient temperatures. Heat exposure before parasitism did not significantly change parasitism rates in any host–parasitoid species combination (Fig. 1B).

### Simulations identify additive effects of heat exposure and parasitism on host mortality

**Fig. 2:**
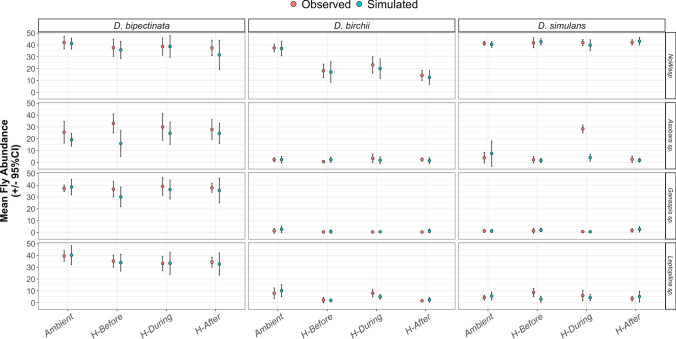
Observed vs. simulated number of flies emerging at the end of the experiments (with 95% confidence intervals). Data are grouped according to host–parasitoid combination and heat exposure timing occurrence. Stars indicate comparisons in which confidence intervals do not overlap and suggest higher order effects. See Online Resource 2 for details on the simulation model

Comparisons between observed and simulated counts of adult emergence are shown in Fig. 2 for *Drosophila* flies and Online Resource 1: Figure S5 for parasitoid wasps. The simulated number of emerging flies approximates the corresponding observed emergences for most treatment combinations, signifying agreement with the null hypothesis that the effects of parasitoid attack and heat exposure timing on host mortality are additive. The only exception is the *D. simulans–Asobara* sp. interaction during heat exposure when significantly more flies hatched than the model predicted, i.e., confidence intervals for observed and expected emergence do not overlap. Thus, higher order effects between the timing of heat exposure and parasitism on host and parasitoid mortalities are generally not supported.

### Heat exposure tolerance of adult flies and wasps

**Fig. 3:**
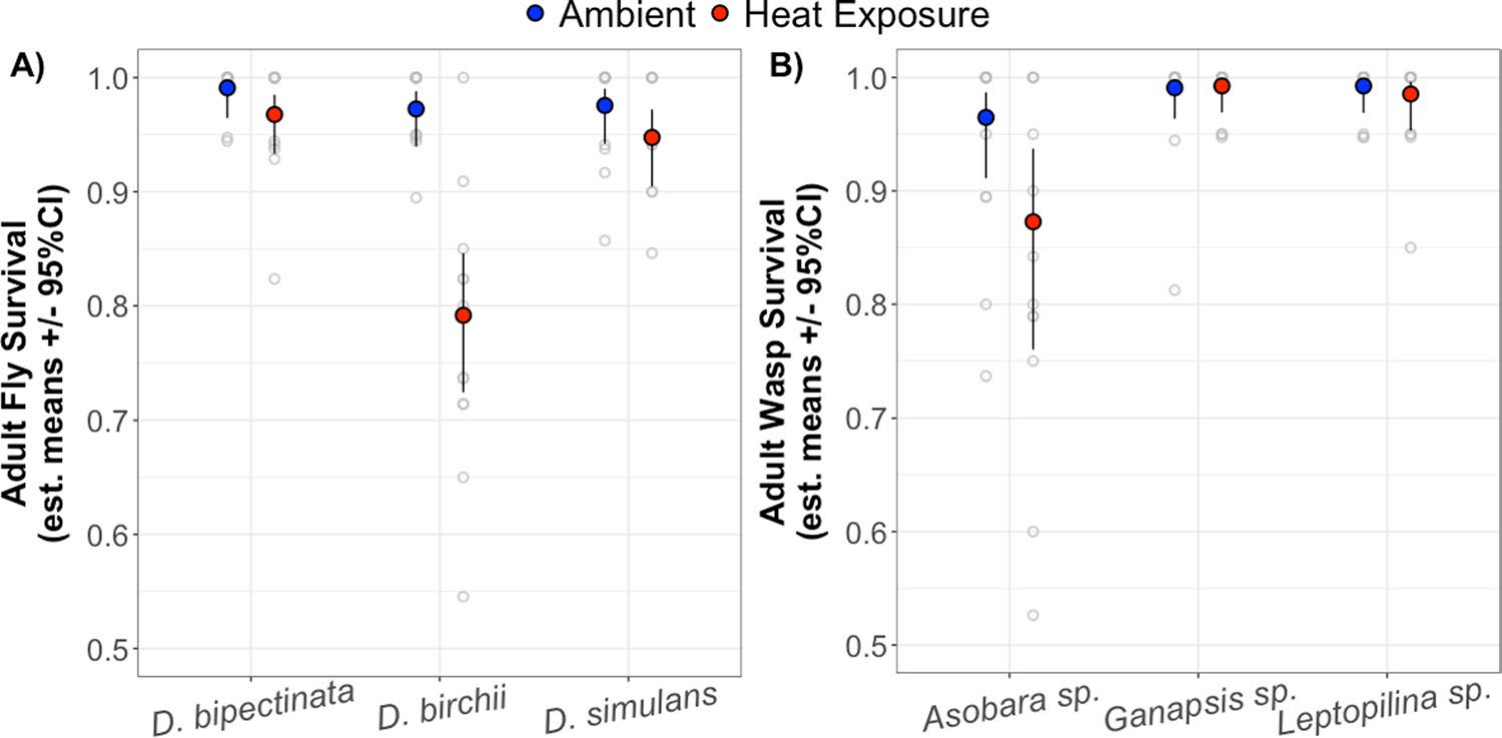
Estimated marginal means in (**A**) adult *Drosophila* host survival and (**B**) adult parasitoid wasp survival, averaged across sexes in extreme heat and ambient temperatures. Grey points are the observed survival rate for each replicate

Responses to a heat exposure were generally weaker for adult flies and wasps than for larvae. The survival of adult *Drosophila* varied significantly among species (F = 12.76, *df* = 2, *p* < 0.001) and in heat exposure (F = 25.53, *df* = 1, *p* < 0.001), but there was no interaction between these effects (Fig. 3A; Online Resource 1: Table S8). Thus, responses to heat exposure differed among species but in similar magnitudes and directions. When averaged across species and sexes, the odds of adult survival following heat exposure were 5.5 times (OR = 0.18, SE = 0.06, *p* < 0.0001) lower than for being exposed to ambient temperatures.

Adult parasitoid survival differed significantly among species (χ^2^ = 21.3, *df* = 2, *p* < 0.0001) and was marginally decreased by heat exposure (χ^2^ = 3.7, *df* = 1, *p* = 0.053). However, there was no interaction between these effects (Fig. 3B; Online Resource 1: Table S9). The marginal trend showed that heat exposure lowered the odds of adult parasitoid survival 2.4 times (OR = 0.412, SE = 0.19, *p* = 0.053).

## Discussion

To predict the impact of extreme heat events on food webs, it is essential to understand how thermal sensitivity varies among species and their different developmental stages. Our results support that *Drosophila* and parasitoid species exhibit variability in their responses to short-term extreme heat exposure. Prior research has also documented differences in thermal tolerance and species distributions among *Drosophila* species within this system (Chen and Lewis 2024). Notably, our findings demonstrate that the impact of a 24-h heat exposure depended on its timing relative to host–parasitoid interactions. Parasitism rates declined significantly in five of six interactions when exposure occurred during or, in one case, after parasitism, but never before. If parasitism coincides with heat exposure, parasitoids may thus become less effective in controlling host populations, potentially leading to significant shifts in natural food webs and increased outbreaks of agricultural pests. Contrary to our expectations, exposure before parasitism did not increase host susceptibility to subsequent parasitism. This was further supported in the simulation model, which demonstrated that the effects of heat exposure and parasitism on host and parasitoid survival were generally additive, and the outcome of the interactions could be predicted based on separate temperature sensitivity and parasitoid resistance tests. Interestingly, qualitative comparisons show adults, especially parasitoids, are more heat-tolerant than immature stages. Adult flies displayed less mortality under extreme heat than larvae. While the success rate of parasitoids developing into adults decreased when larvae and eggs were exposed to extreme heat during or after parasitism, indicating that immature parasitoid stages are less heat-tolerant than adults. The impact of different heat exposure timings on the host was mixed, ranging from negative to positive, but generally proved detrimental to the parasitoids, especially during their early developmental stages. It is important to emphasize that our conclusions are based on host–parasitoid interactions within a microcosm. This context restricted *Drosophila* and parasitoids from locating more favorable microclimatic conditions, potentially making the results more extreme than what happens in natural settings.

### Heat exposure timing impacts parasitism rates

We found that parasitism was significantly reduced when heat exposure occurred during or after parasitism events, while heat exposure that occurred before the parasitism event, exposing early instar larvae, did not affect parasitism rates. The decrease in parasitism did not always lead to higher host survival rates, suggesting that parasitoids still infected hosts at similar or slightly reduced rates. However, fewer of these infections survived to the adult parasitoid stage. Such reduced parasitoid success would likely result in fewer parasitoids available to attack subsequent host generations. Other studies showed that parasitoids may be more active at higher temperatures, increasing super-parasitism rates, where one larval host is parasitized multiple times, but the result was also lower adult emergence success (Thierry et al. 2022). In some cases, lower parasitism rates during heat exposure directly led to increased host survival. In a different study, the emergence of a caterpillar parasitoid was also dramatically reduced when heat exposure occurred on the same day as oviposition (Moore et al. 2021). Our results support other studies that showed that extreme heat events occurring later in an organism’s development or season had the greatest ecological impact and can even be detrimental to adult reproduction in insects (Ma et al. 2004, 2021). For example, Cope et al. (2023) found that heatwaves that occurred later in the growing season of milkweed reduced aphid abundance the most. As extreme heat events start earlier and last longer into the year, they will likely have significant and prolonged effects on ecological communities.

### Trophic levels are affected differently

Our results align with several previous studies, indicating that higher trophic levels, like parasitoids, are more sensitive to brief extreme high temperatures than lower trophic levels (Voigt et al. 2003; Schreven et al. 2017; Moore et al. 2021; Thierry et al. 2021; Malinski et al. 2023). Although there was variation in parasitism rates among species in response to extreme heat, based on successful oviposition, development, and emergence of parasitoids, no parasitoid benefited from heat exposure, regardless of the timing of exposure. Parasitoids are thought to be more vulnerable to extreme temperatures, because increased temperatures may improve host resistance or kill symbiotic micro-organisms employed for successful parasitism (Jeffs and Lewis 2013; Thierry et al. 2019). However, some studies have shown that higher trophic levels were more thermally tolerant (Franken et al. 2018) or benefited from increased temperatures (Bannerman et al. 2011; Gillespie et al. 2012; Barton and Ives 2014), while others showed no change in response to increased temperatures (Hoover and Newman 2004; Sentis et al. 2013).

Fly developmental rates and survival were mainly impacted by heat exposure occurring in late instars. However, egg or early instar larval parasitoids were most vulnerable to heat exposure, indicating that extreme temperatures harmful to eggs or larvae may not affect pupal or adult stages of parasitoids. Studies indicate that the timing of extreme heat is more likely to alter species interactions involving complex, discrete life cycles (i.e., holometabolous insects) than for organisms with more gradual life cycle changes, such as vertebrates (Cinto Mejía and Wetzel 2023). This suggests that the ecological impacts of heat exposure timing on the outcome of species interactions will depend on the individuals’ stage-specific thermal response (i.e., ontogenetic stage) (Ma et al. 2021). For example, in our tropical *Drosophila*–parasitoid community, extreme heat events occurring when there is a high abundance of early instar *Drosophila* may reduce host populations less than when most individuals are late instars. However, parasitoid abundance tends to accumulate over the season, and extreme heat events later in the season will likely harm parasitism success and the parasitoid populations. Diverse ecological communities with different developmental stages that coexist may help stabilize ecosystems experiencing extreme heat events at varying times of the year (Murdoch et al. 1985).

### Combined effects of heat exposure and parasitism are mostly additive

Our mathematical model revealed that the combined effects of heat exposure timing and parasitism are generally additive, suggesting that exposure to heat early in one’s development did not influence host–parasitoid interactions in later developmental stages. We expected that *Drosophila* host species stressed by heat exposure early in development would be weakened and more susceptible to parasitism, thus showing higher rates of parasitism than predicted if effects were additive (Thomas and Blanford 2003; Bensadia et al. 2006). Our results indicate that quantifying the mortalities resulting from different heat exposure timings and parasitism makes it possible to accurately predict the survival rates of hosts and parasitoids using an additive model for most of our species combinations. The lack of synergistic effects of multiple stressors could be due to the stressful temperatures being too short to have lasting effects or compromise the immune response. Studies have shown that early instar insect larvae can restore functionality after being exposed to short, extremely high temperatures (Xing et al. 2014). From the fly perspective, our findings are consistent with the *lifecycle modularity hypothesis* (Potter et al. 2011) and *adaptive decoupling hypothesis* (Moran 1994; Stoks and Córdoba-Aguilar 2012), which propose that modular life cycles can help invertebrates deal with stressful environmental conditions, especially if those conditions occur in the early stages of development. Even though extreme heat events impose stressful conditions on species for brief periods, our research shows that the timing of these events and the species involved can interact, significantly affecting community responses. As extreme heat events become more frequent with climate change, our results highlight the detrimental effects of these brief but intense temperature spikes on parasitoids and their capacity to control lower trophic levels.

## Supplementary Information

Below is the link to the electronic supplementary material.

Supplementary file1 (DOCX 1275 KB)

Supplementary file2 (PDF 236 KB)

Supplementary file3 (M 11 KB)

## Data Availability

All data and R scripts used for this study were archived in Zenodo. 10.5281/zenodo.16799503.

## Code availability

Code for simulation is available as Electronic Supplementary Materials file ESM3_simulation_script.m.
