# Supplementary material for: Timing of extreme heat events matters: exposure during parasitism disrupts top-down control
Source: Oecologia. 2025 Aug 12;207(9):141. doi: 10.1007/s00442-025-05781-6 (PMC12343740; doi:10.1007/s00442-025-05781-6)
Supplement: Supplementary file 1 — Supplementary file1 (DOCX 1275 KB) [file 442_2025_5781_MOESM1_ESM.docx]

**Electronic Supplemental Material 1**

**Oecologia: Original Research**

**Timing of extreme heat events matters: exposure during parasitism disrupts top-down control**

Nicholas A. Pardikes^1,2^, Tomas A. Revilla^1,3^, Gregoire Proudhom^1,3^, Melanie Thierry^1,4^, Chia-Hua Lue^1,5^, and Jan Hrcek^1,3^

1 *Biology Centre of the Czech Academy of Sciences, Institute of Entomology, Ceske Budejovice, Czech Republic*

*2 Department of Biology, Utah State University, Logan, Utah, USA*

*3 Faculty of Science, University of South Bohemia, Ceske Budejovice, Czech Republic*

*4 Centre de Recherche sur la Biodiversité et l'Environnement (CRBE), UMR 5300 CNRS-IRD-TINP-UT3 Université Toulouse III – Paul Sabatier, Toulouse, France*

*5 Department of Biology, Hood College, Frederick, Maryland, USA*

**Supporting figures**

**Figure S1**

**Figure S1:** The temperature conditions for the heat exposure and ambient temperature treatments in each thermal chamber from May 3-7, 2020, are shown as an example.

**Figure S2**

**Figure S2**: The average temperatures for the two growth chamber locations during the experiment (May 10 – Jun 25, 2020). Figure S2 shows the average temperatures for the heat exposure and ambient temperature chambers across the two locations. The mean temperature was 0.36 C colder in the heat exposure chamber in one of our locations, which was a significant difference (+- 0.09 SE, P <0.001).

**Figure S3**

**
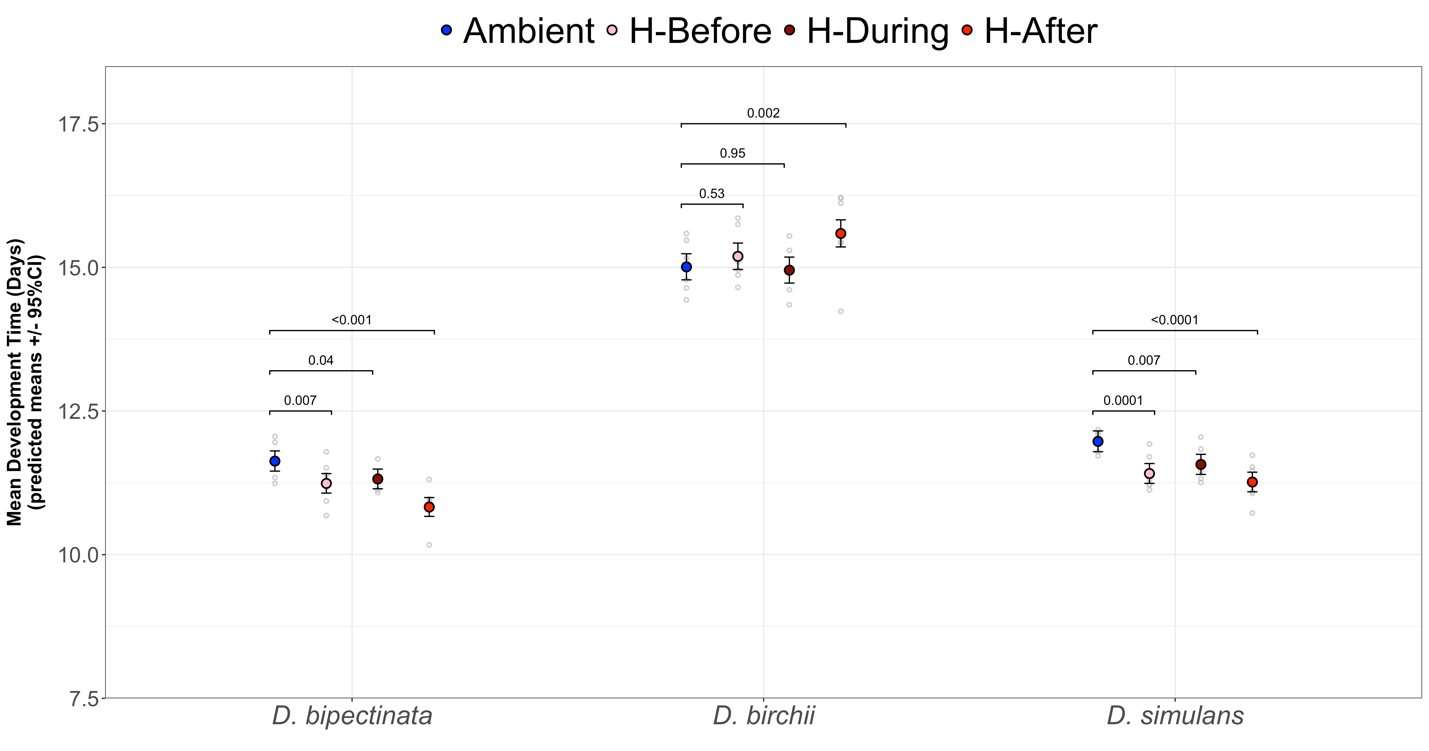
**

**Figure S3:** Drosophila development times under different heat exposure timings without parasitoids. The effect is presented as estimated marginal means in development times averaged across chamber location and block levels. P-values are shown above for each comparison relative to the ambient temperature treatment. Dunnett's method was used to adjust the p-value for multiple comparisons. The tests were performed on the log scale since the response variable was log-transformed.

**Figure S4**


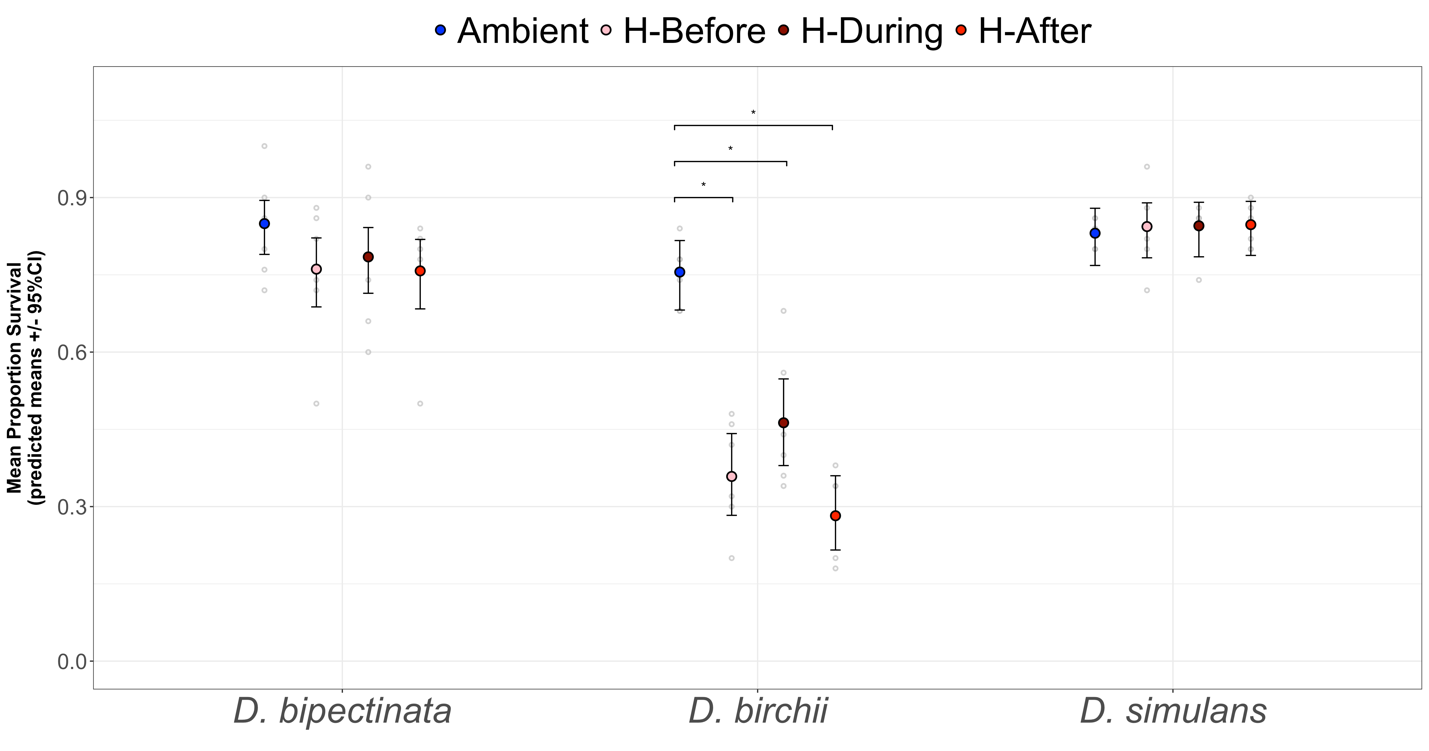


**Figure S4:** Host survival when exposed to different heat timings, and no parasitism. The effect is presented as estimated marginal means in host survival, averaged across chamber location and block levels. Stars indicate significant comparisons (p < 0.05), and the plain black bar represents a marginally significant comparison (p < 0.1) compared to the ambient temperature treatment tested on the log odds ratio scale. The Sidak method was used to adjust for multiple comparisons.

**Figure S5**


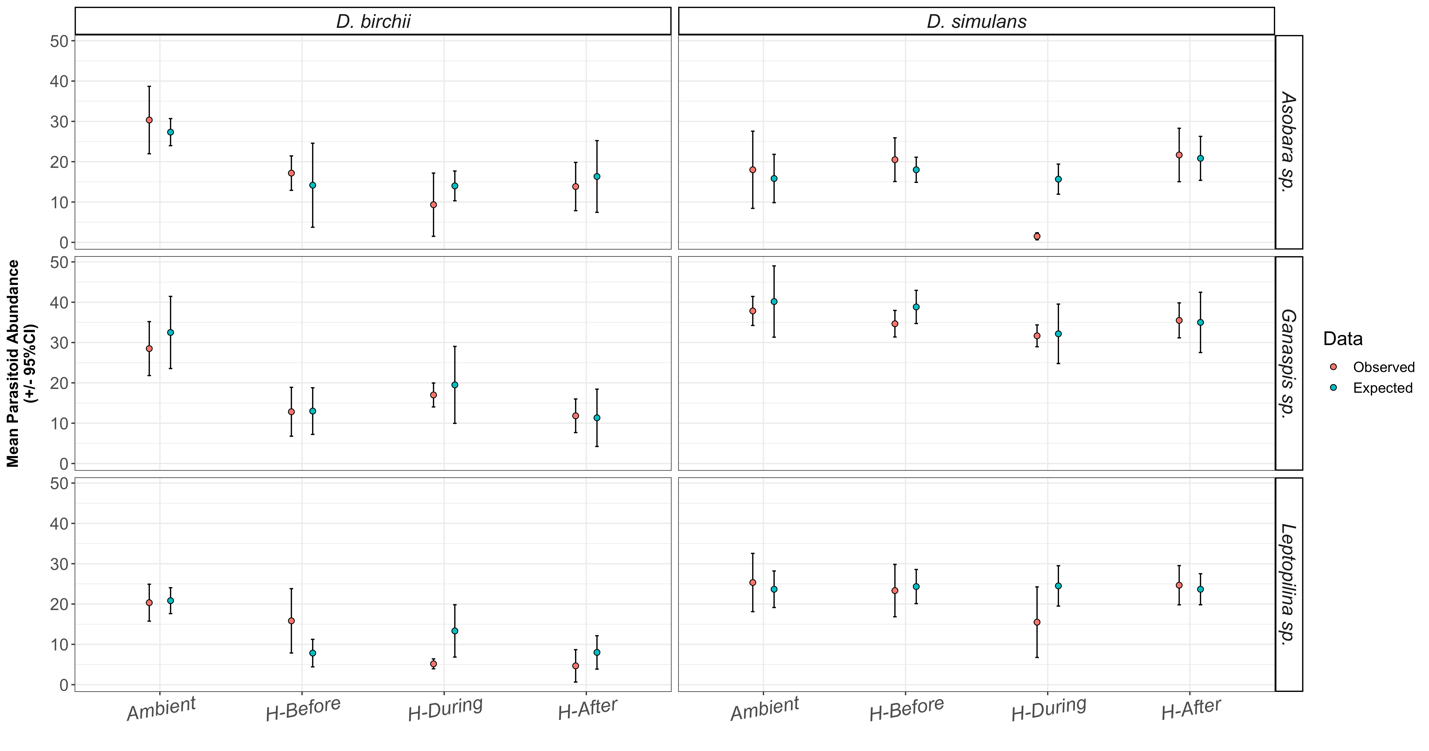


**Figure S5:** Observed vs. simulated (simulation model) number of wasps (95% confidence intervals) emerging at the end of the experiments. Data is grouped according to host-parasitoid combination and heat exposure occurrence. The hosts, *Drosophila*, are listed in panel columns, and the parasitoids are listed in panel rows. The number of replicates is 6 for each category. Results for *D. bipectinata* are omitted as all three parasitoid species experienced total or near total failure developing on this host and results of the model were thus unreliable.

**Supporting Tables**

**Table S1**: Average number of adult flies reared in the absence of parasitoids across all four heat treatments and the standard deviation. Each unique *Drosophila* species and heat combination was replicated six times. The mean fly abundance represents the “T” in all calculations of host survival and parasitism rates.

| ***Drosophila***  **species** | **Heat exposure Treatment** | **Mean Fly Abundance** | **Standard Deviation** |
| --- | --- | --- | --- |
| *D. bipectinata* | Ambient | 42.00 | 5.10 |
| *D. bipectinata* | H-Before | 37.67 | 6.98 |
| *D. bipectinata* | H-During | 38.67 | 6.89 |
| *D. bipectinata* | H-After | 37.50 | 6.28 |
| *D. birchii* | Ambient | 37.50 | 3.15 |
| *D. birchii* | H-Before | 18.17 | 5.42 |
| *D. birchii* | H-During | 23.17 | 6.59 |
| *D. birchii* | H-After | 14.33 | 4.08 |
| *D. simulans* | Ambient | 41.33 | 1.51 |
| *D. simulans* | H-Before | 41.83 | 4.02 |
| *D. simulans* | H-During | 42.00 | 2.53 |
| *D. simulans* | H-After | 42.17 | 2.14 |

**Table S2:** The sum of parasitoid wasp adults reared from each *Drosophila*-parasitoid species combination.

| **Host Species** | **Wasp Species** | **Total Wasps** |
| --- | --- | --- |
| *D. bipectinata* | *Asobara sp.* | 0 |
| *D. bipectinata* | *Ganaspis sp.* | 8 |
| *D. bipectinata* | *Leptopilina sp.* | 0 |
| *D. birchii* | *Asobara sp.* | 424 |
| *D. birchii* | *Ganaspis sp.* | 421 |
| *D. birchii* | *Leptopilina sp.* | 276 |
| *D. simulans* | *Asobara sp.* | 370 |
| *D. simulans* | *Ganaspis sp.* | 838 |
| *D. simulans* | *Leptopilina sp.* | 533 |

**Table S3**: The sum of *Drosophila* adults reared from each *Drosophila*-parasitoid species combination.

| **Host Species** | **Wasp Species** | **Total Flies** |
| --- | --- | --- |
| *D. bipectinata* | *Asobara sp.* | 698 |
| *D. bipectinata* | *Ganaspis sp.* | 905 |
| *D. bipectinata* | *Leptopilina sp.* | 853 |
| *D. birchii* | *Asobara sp.* | 54 |
| *D. birchii* | *Ganaspis sp.* | 18 |
| *D. birchii* | *Leptopilina sp.* | 119 |
| *D. simulans* | *Asobara sp.* | 224 |
| *D. simulans* | *Ganaspis sp.* | 33 |
| *D. simulans* | *Leptopilina sp.* | 135 |

**Table S4:** ANOVA (Type III tests) F-test investigating differences in development times in the different heat exposure timings without parasitoids. Development time was log-transformed to improve model performance.

| **Predictor Variables** | **Sum of squares** | **DF** | **F value** | **P-value** |
| --- | --- | --- | --- | --- |
| *Intercept* | 460.53 | 1 | 1.35E+06 | <0.001 * |
| Treatment | 0.01 | 3 | 9.45E+00 | <0.001 * |
| *Drosophila* Species | 1.32 | 2 | 1.93E+03 | <0.001 * |
| Heat-Location | 0.03 | 1 | 9.29E+01 | <0.001 * |
| Block | 0.01 | 2 | 1.12E+01 | <0.001 * |
| Treatment × *Drosophila* Species | 0.02 | 6 | 1.20E+01 | <0.001 * |
| Residuals | 0.02 | 57 |  | |

**Table S5:** Analysis of Deviance Table (Type III Wald χ^2^ tests) investigating the proportion of hosts surviving to adults when exposed to extreme heat in the absence of parasitoids.

| **Predictor Variables** | **χ^2^** | **DF** | **P-value** |
| --- | --- | --- | --- |
| *Intercept* | 292.34 | 1 | <0.001 * |
| Treatment | 32.83 | 3 | <0.001 * |
| *Drosophila* Species | 208.13 | 2 | <0.001 * |
| Block | 3.96 | 2 | 0.138 |
| Heat-Location | 12.25 | 1 | <0.001 * |
| Treatment × *Drosophila* Species | 38.60 | 6 | <0.001 * |

**Table S6:** Analysis of Deviance Table (Type III Wald χ^2^ tests) for the analysis of host survival when exposed to different heat exposure timings and parasitoids.

| **Predictor Variables** | **χ^2^** | **DF** | **Pr(>χ^2^)** |
| --- | --- | --- | --- |
| *Intercept* | 184.74 | 1 | *<0.001 ** |
| Heat-Treatment | 8.22 | 3 | 0.042 * |
| *Drosophila* Species | 890.57 | 2 | *<0.001 ** |
| Wasp Species | 34.79 | 2 | *<0.001 ** |
| Block | 0.38 | 2 | 0.829 |
| Heat-Location | 2.59 | 1 | 0.108 |
| Heat-Treatment × *Drosophila* Species | 15.42 | 6 | 0.017 * |
| Heat-Treatment × Wasp Species | 26.99 | 6 | *<0.001 ** |
| *Drosophila* Species × Wasp Species | 70.68 | 4 | *<0.001 ** |
| Heat-Treatment × *Drosophila* Species × Wasp Species | 42.88 | 12 | *<0.001 ** |

**Table S7:** Analysis of Deviance Table (Type III Wald χ^2^ tests) for the analysis of parasitism rates when exposed to different timing of heat exposure.

| **Predictor Variables** | **χ^2^** | **DF** | **P-value** |
| --- | --- | --- | --- |
| *Intercept* | 38.57 | 1 | *<0.001* * |
| Heat-Treatment | 85.25 | 3 | *<0.001* * |
| *Drosophila* Species | 0.01 | 1 | 0.946 |
| Wasp Species | 79.86 | 2 | *<0.001* * |
| Block | 0.26 | 2 | 0.880 |
| Heat-Location | 0.56 | 1 | 0.455 |
| Heat-Treatment × *Drosophila* Species | 7.55 | 3 | 0.056 |
| Heat-Treatment × Wasp Species | 27.60 | 6 | *<0.001* * |
| *Drosophila* Species × Wasp Species | 71.64 | 2 | *<0.001* * |
| Heat-Treatment × *Drosophila* Species × Wasp Species | 12.43 | 6 | 0.053 |

**Table S8:** Analysis of Deviance Table (Type III Wald χ^2^ tests) for analyzing adult fly survival rates when exposed to extreme heat. Error estimates are based on Pearson residuals.

| **Predictor Variable** | **Sum of Squares** | **DF** | **F-values** | **P-values** |
| --- | --- | --- | --- | --- |
| Heat-Treatment | 36.79 | 1 | 25.54 | < 0.001 * |
| *Drosophila* species | 36.76 | 2 | 12.76 | < 0.001 * |
| *Drosophila* sex | 1.15 | 1 | 0.80 | 0.374 |
| Residuals | 96.54 | 67 |  |  |

**Table S9:** Analysis of Deviance Table (Type III Wald χ^2^ tests) for analyzing adult parasitoid survival rates when exposed to extreme heat.

| **Predictor Variable** | **χ^2^** | **DF** | **P-value** |
| --- | --- | --- | --- |
| *Intercept* | *157.96* | *1* | *< 0.001 ** |
| Heat-Treatment | 3.74 | 1 | 0.053 |
| Wasp species | 21.35 | 2 | < 0.001 * |
| Wasp sex | 0.078 | 1 | 0.78 |
