# Supplementary material for: Timing of extreme heat events matters: exposure during parasitism disrupts top-down control
Source: Oecologia. 2025 Aug 12;207(9):141. doi: 10.1007/s00442-025-05781-6 (PMC12343740; doi:10.1007/s00442-025-05781-6)
Supplement: Supplementary file 2 — Supplementary file2 (PDF 236 KB) [file 442_2025_5781_MOESM2_ESM.pdf]

# Model for "Timing of extreme heat events matters: exposure during parasitism disrupts top-down control"

Nicholas A. Pardikes, Tomas A. Revilla, Gregoire Proudhom, Melanie Thierry, Chia-Hua Lue, and Jan Hrcek

## 1 Mathematical model

The joint effect of parasitism and extreme heat exposure (EHE) events is modelled by projecting a cohort of hosts "H" over a span of 8 days like in the experiments. Exposure to parasitoid attack at day 3 creates an infected branch "I". At the end of day 8, surviving hosts emerge as flies "F" if uninfected, or as wasps "W" if infected, as illustrated below

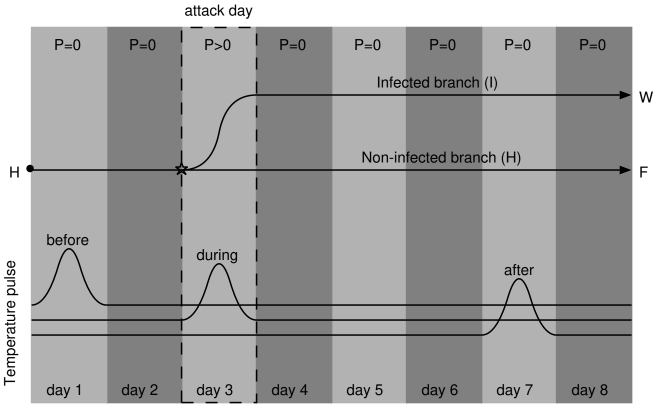


EHE, shown as temperature pulses above, can occur before, during or after parasitoid attack, at day 1, 3 or 7, respectively like in the experiments.

Let $H_t$ and $I_t$ be, respectively, the number of uninfected and infected hosts at the end of day $t = 1, 2 \ldots, 8$. The cohort start with $H_0 = 50$ eggs and $I_0 = 0$ infected hosts, and subsequent numbers are obtained by the recurrences

$$H_{t+1} = H_t e^{-m(t)-a(t)} \tag{1}$$

$$I_{t+1} = I_t e^{-m(t)} + H_t \left(1 - e^{-a(t)}\right) e^{-m(t)}. \tag{2}$$

Hosts survive with with probability $e^{-m(t)}$, $m(t)$ is the *mortality rate* per capita on day $t$ according to

$$m(t) = \begin{cases} m_0 & t \neq \tau \\ m_0 + m_\tau & t = \tau \end{cases}, \tag{3}$$

$m_0$ is a *intrinsic mortality* rate and $m_\tau$ is the *mortality differential* caused by the EHE on day $\tau = 1, 3, 7$. The probability of escaping parasitism on day $t$ is $e^{-a(t)}$ where

$$a(t) = \begin{cases} 0 & t \neq 3 \\ a_0 P & t = 3 \end{cases}, \tag{4}$$

$a_0$ is the attack rate by $P$ parasitoids (3 females). The non escaping fraction $1 - e^{-a(t)}$ is used to increment the number of infected hosts.

At the end of day $t = 8$, uninfected hosts emerge as $F = H_8$ adult flies. $W = \varepsilon I_8$ parasitoids emerge from infected hosts which die, where $\varepsilon$ is the conversion rate of wasps per infected host. Experiment data provides numbers of flies $F$ and wasps $W$ emerging on day 8 and after. Substituting (3, 4) in system (1, 2) and integrating from $t = 0$ to $T = 8$ gives

$$F = H_0 e^{-m_0 T - m_\tau - a_0 P} \tag{5}$$

$$W = \varepsilon H_0 e^{-m_0 T - m_\tau} \left(1 - e^{-a_0 P}\right). \tag{6}$$

Our model assumes that there is no interaction between attack rates, mortality and mortality differentials, e.g., $m_0$ and $m_\tau$ values are independent of the presence of parasitoids, and $a_0$ values are independent of EHE, before, during or after. This independence (*null hypothesis*) can be tested by comparing empirical outcomes with stochastic simulations based on host dynamics (1, 2). The comparissons require estimation of $m_0, m_\tau, a_0$ and $\varepsilon$.

# 2 Parameter estimation

Experiment data is used to calculate empirical mortality per host per day using the formula

$$\mu = \frac{1}{T} \ln\left(\frac{H_0}{F}\right) \approx \frac{2.3026}{T} \log_{10}\left(\frac{H_0}{F}\right). \tag{7}$$

There was small fraction of experiments where all hosts got infected and $F = 0$. For these cases we set $F = 1$ in order to perform calculations. Thus $\mu$ overestimates real pre-adult mortality rates. According to (5), daily mortality follows the linear model

$$\mu = m_0 + \frac{m_\tau}{T} + \frac{a_0 P}{T}, \tag{8}$$

which facilitates parameter estimations. Intrinsic mortality rate $m_0$ corresponds to mortality recorded from double control experiments

$$m_0 = \mu_{00},$$

i.e., without EHE (1st sub-index is 0) and without parasitoids (2nd sub-index is 0). Mortality differentials $m_\tau$ are obtained using difference between the mortality with EHE minus mortality in the double control (no EHE and no parasitoids)

$$m_\tau = (\mu_{\tau 0} - \mu_{00})T, \tag{9}$$

and $a_0$ is obtained using the difference between the mortality under attack without EHE, minus mortality in the double control

$$a_0 = (\mu_{03} - \mu_{00})T/P. \tag{10}$$

According to equation (6) the number of emerging wasps without EHE is

$$W_{03} = \varepsilon_0 \left(He^{-m_0 T} - He^{-m_0 T - a_0 P}\right) = \varepsilon_0 \left(F_{00} - F_{03}\right)$$

where the quantity $He^{-m_0 T}$ is expected to match the empirical number of flies $F_{00}$ that emerge under the double control (no EHE and no parasitoids), whereas $He^{-m_0 T - a_0 P}$ is expected to match the empirical number of flies $F_{03}$ that emerge if parasitoid attack happened but EHE didn't. From this we get

$$\varepsilon = \frac{W_{03}}{F_{00} - F_{03}}, \tag{11}$$

Since $m_0$, $m_\tau$'s, and $a_0$ are linear related, it is very easy to derive expected values and variances

$$
\begin{aligned}
E[m_0] &= E[\mu_{00}] \\
V[m_0] &= V[\mu_{00}] \\
E[m_\tau] &= T\left(E[\mu_{\tau 0}] - E[\mu_{00}]\right) \\
V[m_\tau] &= T^2\left(V[\mu_{\tau 0}] + V[\mu_{00}]\right) \\
E[a_0] &= T\left(E[\mu_{03}] - E[\mu_{00}]\right)/P \\
V[a_0] &= T^2\left(V[\mu_{03}] + V[\mu_{00}]\right)/P^2
\end{aligned}
$$

where $T^2 = 8^2 = 64$, $P^2 = 3^2 = 9$. The experiments from which the $\mu$'s are sourced are independent, so any potential covariances are implied to be zero. In practice, $\mu$'s expectations and variances are given by corresponding sample means and sample variances.

For the host to wasp conversion rate $\varepsilon$ it is not possible obtain closed formulas for their variances because the relations between $W$'s and $F$'s are not linear. Instead, we estimate $\varepsilon$ using sample averages $\bar{W}_{03}, \bar{F}_{00}, \bar{F}_{03}$ in (11).

# 3 Simulation

We implement the following stochastic simulation protocol

1. Set $H_0 = 50$ and $I_0 = 0$
2. From day $t = 1$ to day $T = 8$ calculate
   a) Deaths: draw $m$ from a normal distribution with mean $E[m_0]$ and variance $V[m_0]$. If an EHE occurs, add a mortality differential drawn from a normal distribution with mean $E[m_\tau]$ and variance $V[m_\tau]$, with $\tau = t$. For each host $i = 1, \ldots, H + I$, draw $x_i$ uniformly between 0 and 1: if $x_i > e^{-m}$, subtract 1 from $H$ or from $I$, which one depends whether $i$ is non-infected or infected respectively.
   b) Attacks: set $a$ equal to $P = 3$ (female wasps) times a number drawn from a normal distribution with mean $E[a_0]$ and variance $V[a_0]$. For each non-infected host $i = 1, \ldots, H$, $x_i$ is drawn from a uniform distribution between 0 and 1, if $x_i > e^{-a}$ then subtract 1 from $H$ and add 1 to $I$.
3. Set $F = H_8$ and draw $W$ from a Poisson distribution with parameter $\varepsilon I_8$.

This is protocol applies to each combination of: (i) host species; (ii) parasitoid species, including no parasitoid; and (iii) timing of EHE (including no parasitoid and/or no EHE as controls). The number of replicas for each combination matches the corresponding data sample size. The 95% confidence intervals for number of emerging adult flies $F$ and wasps $W$ are visually compared with corresponding 95% confidence intervals from the data.

The simulations were run using Matlab R2024a. All necessary commands are listed and executed by the single m-file: `simulation_script.m`
